# Physical Fitness and Chemotherapy Tolerance in Patients with Early-Stage Breast Cancer

**DOI:** 10.1249/MSS.0000000000002828

**Published:** 2021-12-27

**Authors:** Wim G. Groen, Willeke R. Naaktgeboren, Wim H. van Harten, Jonna K. van Vulpen, Nathalie Kool, Gabe S. Sonke, Elsken van der Wall, Miranda J. Velthuis, Neil K. Aaronson, Anne M. May, Martijn M. Stuiver

**Affiliations:** 1 The Netherlands Cancer Institute, Division of Psychosocial Research and Epidemiology, Amsterdam, the NETHERLANDS; 2 Julius Center for Health Sciences and Primary Care, University Medical Center Utrecht, Utrecht University, Utrecht, the NETHERLANDS; 3 Rijnstate Hospital, Arnhem, the NETHERLANDS; 4 Department of Health Technology and Services Research, University of Twente, Enschede, The NETHERLANDS; 5 Department of Radiation Oncology, University Medical Center Utrecht, Utrecht, the NETHERLANDS; 6 School of Physiotherapy, Faculty of Health, Amsterdam University of Applied Sciences, Amsterdam, the NETHERLANDS; 7 Department of Medical Oncology, the Netherlands Cancer Institute, Amsterdam, the NETHERLANDS; 8 Department of Medical Oncology, UMC Utrecht, the NETHERLANDS; 9 Netherlands Comprehensive Cancer Organisation, Utrecht, the NETHERLANDS; 10 Center for Quality of Life, The Netherlands Cancer Institute, Amsterdam, the NETHERLANDS; 11 Centre of Expertise Urban Vitality, Faculty of Health, Amsterdam University of Applied Sciences, Amsterdam, the NETHERLANDS; 12 Department of Rehabilitation Medicine, Amsterdam University Medical Centers, Vrije Universiteit Amsterdam, Cancer Center Amsterdam, Amsterdam, the NETHERLANDS

**Keywords:** BREAST CANCER, CHEMOTHERAPY, CLINICAL CANCER RESEARCH, PHYSICAL FITNESS

## Abstract

**Introduction:**

An optimal relative dose intensity (RDI) of adjuvant chemotherapy is associated with better survival in patients with breast cancer. Little is known about the role of physical fitness in attaining an adequate RDI in patients with early-stage breast cancer. We investigated the association between pretreatment physical fitness and RDI in this population.

**Methods:**

We pooled individual patient data from two randomized exercise trials that studied exercise programs in early breast cancer: the Physical Exercise During Adjuvant Chemotherapy Effectiveness Study (*n* = 230) and the Physical Activity during Chemotherapy Treatment (*n* = 204) study. Logistic regression models were used to evaluate the association between pretreatment fitness and achieving an optimal RDI (≥85%). In addition, we added an interaction term to the model to explore the potential moderating effect of participating in an exercise program.

**Results:**

Data were available for 419 patients (mean age at diagnosis, 50.0 ± 8.6 yr). In the total sample, lower pretreatment physical fitness was associated with significantly lower odds of achieving ≥85% RDI: age-adjusted odds ratio (OR) of 0.66 (95% confidence interval (CI), 0.46–0.94). In patients allocated to the supervised exercise intervention during chemotherapy (*n* = 173), the association between pretreatment physical fitness and RDI was almost completely mitigated (OR, 0.95 (95% CI, 0.54–1.56)), whereas it was more pronounced in patients who received care as usual (*n* = 172; OR, 0.31 (95% CI, 0.13–0.63); *P*_interaction_ = 0.022).

**Conclusions:**

Early-stage breast cancer patients with relatively lower levels of pretreatment physical fitness have lower odds of achieving an optimal dose of chemotherapy. Given that physical fitness is modifiable and our results suggest that following a moderate-to-high intensity exercise training during chemotherapy could improve treatment completion, clinicians should not refrain from referring patients to supportive exercise programs because of low fitness.

Over the past decades, 5-yr breast cancer survival rates have continued to improve and are currently higher than 90% in the Netherlands for stage I and II breast cancer (1). Improvement in chemotherapy is considered as one of the key elements that have contributed to this increased survival rate (2). The amount of chemotherapy received is often expressed as relative dose intensity (RDI), which is the ratio of the actual versus the planned dose intensity (DI). In the adjuvant setting, an RDI of 85% is a widely accepted threshold, as patients who achieve this threshold have a greater likelihood of improved outcomes, including recurrence-free and overall survival (3). Failure to achieve the 85% RDI is reported in over a quarter of breast patients, mostly due to toxicity, even in the current era of adequate supportive care (e.g., effective antiemetics) and tailored treatment regimens (4). In a study of more than 10,000 breast cancer patients treated with contemporary chemotherapy regimens, dose delay ≥7 d or dose reduction of ≥15% was observed in 36% and 35% of the cases, respectively (3). Hence, strategies to increase the likelihood of achieving 85% RDI in this patient population are warranted.

Currently, breast cancer patients at risk of not completing their planned chemotherapy treatment are the elderly and those with poor performance status (3). There is also some evidence that pretreatment low lean body mass (5,6) and low self-reported exercise levels are related to decreased chemotherapy completion rates (7), whereas exercise during chemotherapy might have a beneficial effect on treatment tolerance. The latter was observed in the randomized Physical Exercise During Adjuvant Chemotherapy Effectiveness Study (PACES), in which dose adjustments occurred less frequently in the exercise groups compared with the controls (8). Recently, first evidence was provided that pretreatment physical fitness was associated with better chemotherapy tolerance (9). Whether subsequent participation in an exercise program modifies this association has not been investigated.

Therefore, we conducted a secondary analysis of data from two randomized controlled trials, including PACES, that evaluated the effects of a supervised exercise program during adjuvant chemotherapy for early-stage breast cancer. We assessed the association between pretreatment physical fitness and completing chemotherapy treatment (attaining >85% RDI). In addition, we explored whether participating in an exercise program modifies this association.

## METHODS

### Setting and participants

Data from the Physical Activity during Chemotherapy Treatment (PACT) study and PACES were used for the current analysis. The study design and results of the PACT study (10–12) and PACES (8,13) have been published elsewhere. In brief, the original multicenter studies were both conducted in the Netherlands between 2009 and 2013 and investigated the effect of an exercise program during adjuvant chemotherapy on fatigue, cardiorespiratory fitness, quality of life, and further secondary outcomes. In the PACT study, breast cancer patients were randomly allocated to either an 18-wk moderate-to-high-intensity, supervised exercise program (*n* = 102) or a usual care (UC) control group (*n* = 102). PACES had two intervention groups and a UC control group (*n* = 77). The interventions in PACES were a low-intensity, home-based exercise program (*n* = 77) and a moderate-to-high intensity supervised exercise program (*n* = 76). The latter was rather similar to PACT’s intervention, both comprising two combined aerobic and resistance exercise sessions per week. In addition, participants allocated to these study arms were asked to be physically active for at least 30 min·d^−1^ for 5 d·wk^−1^. In both studies, adherence to the exercise program was recorded by case report files. The attendance rates for the supervised exercise sessions were 83% and 71% in PACT and PACES, respectively (8,11). In PACT, the intervention started within 6 wk after diagnosis with a fixed duration of 18 wk. The PACES interventions started before chemotherapy and continued until 3 wk after chemotherapy. Inclusion criteria for PACT and PACES were comparable and comprised a histological diagnosis of early breast cancer; being scheduled for adjuvant chemotherapy; having no contraindications for physical activity in terms of malnutrition, serious orthopedic, cardiovascular, or pulmonary diseases; and having basic fluency in the Dutch language. For PACT, patients had to be age between 25 and 75 yr, whereas PACES did not have any age restrictions. Patients were excluded from PACT if they had a Karnofsky performance status score <60. All subjects provided written informed consent, and the PACT and PACES studies were approved by the institutional review boards of the University Medical Center Utrecht and The Netherlands Cancer Institute respectively.

### Study measures

Treatment data were extracted from the medical records. This included planned and actually administered chemotherapy (type, dose, and duration). For each agent, both the planned and actual DI were calculated by dividing the total cumulative dose, expressed in milligrams per meter squared body surface area, by treatment duration in weeks (14). These analyses were limited to chemotherapeutic agents and thus not incorporate the usage of monoclonal antibodies (i.e., trastuzumab). Treatment duration was calculated as the duration between the first day of chemotherapy administration and the day of completion of the last cycle. RDI was calculated by dividing actual DI by planned DI and was expressed as a percentage. An overall RDI per regimen was calculated by averaging the RDI of all agents included in that regime, regardless whether agents were given simultaneously or as a sequential drug combination (14). In case a switch from one type of chemotherapy to another occurred, RDI for the first and remaining part of the new regimen was calculated separately and averaged to obtain one RDI per patient.

Physical fitness was assessed in PACT and PACES before randomization and after the exercise intervention had been completed. In the PACT study, a cardiopulmonary exercise test with continuous breathing gas analysis was used, where cycling workload was increased every minute by 10, 15, or 20 W till exhaustion, a symptom limitation, or at the discretion of the supervising physician. Peak oxygen uptake (V̇O_2peak_) was defined as the average value for the last 30 s before exhaustion and was expressed in milliliters per kilogram per minute. In PACES, physical fitness was assessed with a Steep Ramp Test. After a 3-min warm-up at 10 W, resistance increased by 25 W per 10 s until exhaustion and until the revolutions per minute dropped below 60 despite strong verbal encouragement. The outcome of this test, the maximum short exercise capacity (MSEC), is defined as the highest workload achieved before patients can no longer maintain a cadence >60 rpm. A more detailed description of both tests is provided in the protocol papers of the original studies (10,13). The outcomes of the cardiopulmonary exercise test (V̇O_2peak_) and Steep Ramp Test (MSEC) have been shown to be highly correlated (ranging from 0.73 to 0.86) in healthy and patient populations including cancer survivors (15–20).

### Statistical analyses

A binary threshold of 85% RDI per chemotherapy regimen was used as the outcome variable. This was chosen on the basis of the predictive value of this threshold in terms of overall survival in the adjuvant treatment setting (21). Baseline characteristics were computed for the overall cohort and expressed as means (SD) or frequencies (percentages).

Measurements for fitness, expressed either as V̇O_2peak_ (PACT) or MSEC (PACES), were converted into *z* scores by subtracting the mean and then dividing by the SD. Binary logistic regression models were used with *z* scores for fitness as the explanatory variable and RDI (<85%/≥85%) as the dependent variable. Potential confounders for these analyses were defined *a priori* using directed acyclic graphs (22) and included age, body mass index, presence or absence of comorbidities, breast cancer subtype (triple negative; HR+/Her2Neu−; HR−/Her2Neu+, HR+/Her2Neu+). Potential confounders were only included as covariates in the analyses if they were associated with both the explanatory and the outcome variables in the data, as based on the point estimates of association regardless of statistical significance, and changed the estimate of the odds ratio (OR) for the central determinant by >10% when added to the model (23). All models were adjusted for study (PACT or PACES). A nonlinear term (restricted cubic spline) was used to investigate a possible threshold effect of physical fitness on 85% RDI%.

To explore whether participation in a moderate-to-high-intensity exercise program modifies the association between pretreatment fitness and 85% RDI, we added an interaction term to the adjusted model. All exercise analyses were on an intention-to-treat basis and limited to the moderate-to-high-intensity supervised exercise and UC groups only, because the home-based exercise group of PACES was too small. ORs and their 95% confidence intervals (95% CI) were calculated for each group, including the low-intensity, home-based group of PACES, separately (the latter only for exploratory purpose).

All data were analyzed with R (version 3.4.3) and Rstudio software (Version 1.2.5001; Rstudio Inc., Boston, MA). A two-sided *P* value of 0.05 was considered statistically significant.

## RESULTS

### Participants

A total of 434 breast cancer patients participated in PACT or PACES, of whom 22 were excluded from the current analysis because of the absence of sufficient information on chemotherapy regimen (*n* = 10) or because no baseline fitness test had been performed (*n* = 5). Characteristics of the total sample (*n* = 419) are presented in Table 1.

**TABLE 1:** Baseline characteristics of the combined study of the PACT and PACES studies of patients with breast cancer receiving adjuvant chemotherapy.

	All Patients	
	(*n* = 419)	
	Mean	SD
Age (yr)	50.0	8.6
Height (m)	168.4	6.5
Weight (kg)	73.8	13.7
BMI (kg·m^−2^)	26.0	4.6
MSEC (W; *n* = 222)*^a^*	255.4	48.9
V˙O_2peak_ (mL·min^−1^·kg^−1^; *n* = 197)*^b^*	23.8	5.25
	**Pct.**	***n***
Original study		
- PACT	47.0	197
- PACES	53.0	222
Study arm		
- PACT: intervention	23.9	100
- PACT: control	23.2	97
- PACES: supervised, high-intensity	17.4	73
- PACES: home-based, low-intensity	17.6	74
- PACES: care as usual	17.9	75
Presence of comorbidities (%)	22.0	92
T stage		
- 1	55.4	232
- 2	38.9	163
- 3	4.1	17
- 4	1.0	4
- Missing	0.7	3
N class		
- 0	43.9	184
- 1	46.8	196
- 2	6.7	28
- 3	2.6	11
Receptor status		
- Triple negative	17.4	73
- HER+, ER, or PR+	17.2	72
- HER+, ER, or PR−	5.5	23
- HER−, ER, or PR+	60.0	250
Type of chemotherapy		
- TAC	32.9	13
- FEC or AC	14.3	60
- AC/EC, followed by taxanes	26.3	110
- 3 FEC + docetaxel	23.4	98
- Other	3.1	11

*^a^*Only for PACT participants.

*^b^*Only for PACES participants.

AC, doxorubicin, cyclophosphamide; BMI, body mass index; FEC, 5-FU, epirubicin, cyclophosphamide; TAC, docetaxel, doxorubicin, cyclophosphamide.

The most frequently administrated chemotherapy regimen was the combination of docetaxel, doxorubicin, and cyclophosphamide, followed by a sequential treatment regimen that comprises an anthracycline (doxorubicin or epirubicin) and cyclophosphamide, followed by either docetaxel or paclitaxel. More detailed information on chemotherapy regimens is provided in Supplemental Digital Content 1 (see Table, Supplemental Digital Content, chemotherapy regimens, http://links.lww.com/MSS/C454).

In total, 43 patients (10.3%) did not achieve ≥85% RDI (Table 2). Most common reasons for poor chemotherapy tolerance were neuropathy (*n* = 11; 25.6%), nausea and/or vomiting (*n* = 5; 11.6%), myelosuppression (*n* = 4; 9.3%), cardiac signs and/or symptoms (*n* = 4; 9.3%), and malaise (*n* = 4; 9.3%). For *n* = 8 (18.6%), the specific reason for dose modification was not reported.

**TABLE 2:** Reasons for not achieving ≥85% RDI of the chemotherapy regime as planned.

	*n*	Pct.
Neuropathy	11	25.6%
Nausea and/or vomiting	5	11.6%
Myelosupression	4	8.3%
Cardiac signs and/or symptoms	4	8.3%
Malaise	4	8.3%
Own initiative	3	7.0%
Febrile neutropenia	3	7.0%
Gastro-intestinal symptoms	1	2.3%
Unknown	8	18.6%
Total	43	100.0

### Association between fitness and RDI

When adjusted for study, lower pretreatment physical fitness was associated with lower odds of achieving ≥85% RDI: OR of 0.60 (95% CI, 0.42–0.84). There was no indication of nonlinearity (*P* = 0.80), suggesting no threshold effect. Of the possible confounders assumed within the causal model, only age was associated with both determinant and outcome. When correcting for age and study, low pretreatment physical fitness remained associated with not achieving RDI ≥85%: OR of 0.66 (95% CI, 0.46–0.94).

Participation in a supervised exercise program significantly modified the association between baseline fitness and RDI ≥85% (*P*_interaction_ = 0.022). In subsequent stratified analyses, for participants of the moderate-to-high intensity supervised exercise program (*n* = 173) and the low-intensity home-based exercise program of PACES (*n* = 74), pretreatment physical fitness was not associated with an RDI ≥85%: OR of 0.95 (95% CI, 0.54–1.56) and OR of 0.88 (95% CI, 0.38–2.09), respectively. In contrast, in patients allocated to the UC groups (*n* = 172), the association between lower pretreatment physical fitness and not reaching RDI ≥85% was more pronounced: OR of 0.31 (95% CI, 0.13–0.63; Table 3).

**TABLE 3:** The association between baseline physical fitness and not achieving ≥85% RDI of the chemotherapy regime as planned.

Overall Analysis	*n* Included in Analysis	OR	95% CI
Baseline physical fitness	419	0.66	0.46–0.94
Stratified analyses per randomization*^a^*			
Supervised, moderate-to-high-intensity exercise program	173	0.95	0.54–1.56
Home-based, low-intensity exercise program*^b^*	77	0.88	0.38–2.09
Care as usual	169	0.31	0.13–0.63

All presented results are adjusted for age and study (PACT vs PACES).

*^a^*Participation in a supervised, moderate-to-high exercise program moderates the association between baseline physical fitness and RDI ≥85% (*P*_interaction_ = 0.022).

*^b^*This group consists of PACES participants only.

## DISCUSSION

In the present study, we found that breast cancer patients with lower pretreatment physical fitness had a lower likelihood of completing chemotherapy as planned. Accordingly, assessing pretreatment physical fitness could aid in identifying those at risk for not completing chemotherapy. This subgroup of patients is in need of supportive care and might benefit from an exercise program. Our explorative analysis supports the idea that a moderate-to-high-intensity exercise program might mitigate the association between low pretreatment physical fitness and not achieving sufficient RDI. Although the current evidence for the effectiveness of exercise programs to improve treatment completion is inconclusive, with few other options available to improve physical fitness, and considering that exercise is safe for cancer patients (24) and has many positive effects on chemotherapy-related symptoms (e.g., fatigue) (24), referral to an exercise program could be considered, even, or maybe especially, for patients with lower pretreatment fitness.

When considering the known association between attaining at least 85% RDI and efficacy of chemotherapy in terms of survival and disease progression, the findings of this study point out the importance of pretreatment fitness. Recently, it was shown that patients with RDI <85% have a 38% increased risk of dying from breast cancer compared with those with RDI ≥85%. (25) Accordingly, sufficient baseline fitness, or after an exercise program during chemotherapy to mitigate the risk of not achieving 85% RDI due to compromised baseline fitness, can be related to improved survival for early-stage breast cancer patients. Indeed, Courneya et al. (26) found, in an exploratory follow-up analysis (median of 7.5 yr) of their randomized exercise trial during chemotherapy, that disease-free survival tended to be higher in patients who had been allocated to an exercise group during treatment, as compared with those who were allocated to the control group (disease-free survival, 82.7% vs 75.6%, respectively; hazard ratio (HR), 0.68; 95% CI, 0.37–1.24). Hayes et al. (27) found similar HR for disease-free survival in their follow-up of two exercise trials (HR, 0.66, 95% CI, 0.38–1.17; *P* = 0.16). Although these studies are clearly underpowered for such analyses, they show consistent results.

Our findings that higher pretreatment physical fitness is associated with a lower risk of dose modifications is in line with a recently published study (9). This secondary analysis of the previously conducted START and CARE study showed that breast cancer patients in the highest 20% versus lowest 80% of absolute V̇O_2peak_ were approximately two times more likely to achieve 85% RDI (9). Given that this analysis included breast cancer patients recruited between 2002–2005 (START) and 2008–2011 (CARE), our results complement this study by demonstrating that, in women treated with contemporary chemotherapy regimens where chemotherapy tolerance is higher (~80% vs ~90% achieved RDI ≥85% respectively), pretreatment physical fitness remains a significant factor associated with chemotherapy completion. Moreover, we found that physical exercise, and specifically exercise with a moderate-to-high intensity, modified the association between pretreatment physical fitness and chemotherapy tolerance, suggesting that the subgroup of patients with lower pretreatment physical fitness might benefit from referral to an exercise program.

Our finding that patients with relatively low physical fitness have lower odds of completing chemotherapy may be, at least to some extent, related to the amount and quality of skeletal muscle mass. In patients with breast cancer, it has been shown that a higher relative lean mass is associated with a lower risk of chemotherapy modifications (5) and that skeletal muscle gauge (product of muscle quantity and quality) is associated with severe toxicities and hospitalization (6). Similar results have also been reported for colorectal cancer patients (28,29). Nevertheless, in a recent pooled analysis of two exercise trials (*n* = 543 breast cancer patients), body composition, including lean body mass, was not found to be associated with chemotherapy tolerance (9). The authors speculate that their relative fit and healthy study sample could explain this discrepancy in results. Further studies are warranted to document whether standard chemotherapy dosing to body surface area, compared with lean mass, is more likely to result in toxicities in patients with relatively low lean body mass. It has been proposed that standard chemotherapy dosing in relation to body surface area, compared with lean mass, may more easily lead to toxicities in patients with relatively low lean body mass. In addition, endurance exercise may protect the muscle from anthracycline-induced atrophy (30–32), but it is currently unknown if this relates to better chemotherapy completion rates.

In the explorative analysis, we found that exercise might counteract the increased risk of compromised pretreatment physical fitness. To date, few exercise trials have analyzed chemotherapy completion, and these show mixed results. A systematic review concluded that evidence is not sufficient to affirm that exercise has an effect on chemotherapy completion rate (33). This was corroborated by a more recent analysis by Mijwel et al. (32) of the OptiTrain study, in which no beneficial effect of aerobic nor resistance training on chemotherapy completion was found. Also, Kirkham et al. (34) found no difference in frequency of dose adjustments for the total sample of breast cancer patients in their nonrandomized study comparing combined strength and endurance exercise with historical controls who received care as usual. They did, however, find significantly less dose adjustments for regimens containing doxorubicin in the exercise group (34). Nonetheless, in the reported studies, chemotherapy completion was a secondary outcome. In addition, pretreatment physical fitness was not incorporated in any of these analyses, and it is conceivable that participants of these trials were relatively healthy (35). Therefore, in light of our results indicating that those with lower pretreatment physical fitness levels are more likely to benefit from an exercise program, future studies with chemotherapy completion as a primary outcome and a representative study population for the breast cancer population as whole are required to pertain an effect of exercise on chemotherapy completion.

The major strength of our study lies in the availability of a large patient sample, derived from randomized controlled trials, thereby providing detailed information on physical fitness and chemotherapy data. Although our results are based on secondary analyses of two different studies, we adjusted for study in our analyses and we used *z* scores for the outcomes of interest.

As limitations, we would note that home-based exercise group of the PACES trial was relatively small prohibiting a proper dose–response analysis for that subgroup. Also, the two different yet highly correlated measures were used to assess physical fitness in the two studies: V̇O_2peak_ (PACT) or MSEC (PACES). The impact on our analyses was, however, limited by using a *z* score for physical fitness measurements and adjusting our analyses for original study participation (PACT vs PACES). Furthermore, our data need to be interpreted with some caution, given the fact that the confidence intervals around the ORs for both the high- and low-intensity exercise groups overlap slightly with those of the UC groups. Last, we cannot rule out the possibility that clinicians selected chemotherapy regimens according to pretreatment physical fitness (i.e., those with lower fitness receive less intense regimens), which could have diluted our results.

## CONCLUSIONS

Despite these cautionary remarks, the results of our study clearly suggest that in patients with early-stage breast cancer, a lower level of physical fitness at the start of adjuvant chemotherapy is associated with a higher risk of not attaining 85% RDI, thereby compromising long-term patient outcome. Physical exercise while receiving chemotherapy, and specifically exercise with moderate to high intensity, might mitigate this association. Hence, assessing pretreatment physical fitness is of importance to identify those patients at risk for not completing chemotherapy and who might therefore gain additional benefit from an exercise program as supportive care.

## Supplementary Material

## References

[1] Netherlands Comprehensive Cancer Organisation. Available from: www.iknl.nl/kankersoorten/borstkanker/registratie/overleving. Accessed August 20, 2020.

[2] Rossi L Stevens D Pierga JY, . Impact of adjuvant chemotherapy on breast cancer survival: a real-world population. *PLoS One*. 2015;10(7):e0132853. 26214853 10.1371/journal.pone.0132853 PMC4516355

[3] Lyman GH. Impact of chemotherapy dose intensity on cancer patient outcomes. *J Natl Compr Canc Netw*. 2009;7(1):99–108. 19176210 10.6004/jnccn.2009.0009

[4] Denduluri N Patt DA Wang Y, . Dose delays, dose reductions, and relative dose intensity in patients with cancer who received adjuvant or neoadjuvant chemotherapy in community oncology practices. *J Natl Compr Canc Netw*. 2015;13(11):1383–93. 26553767 10.6004/jnccn.2015.0166

[5] van den Berg MMGA Kok DE Posthuma L, . Body composition is associated with risk of toxicity-induced modifications of treatment in women with stage I–IIIB breast cancer receiving chemotherapy. *Breast Cancer Res Treat*. 2019;173(2):475–81. 30353244 10.1007/s10549-018-5014-5 PMC6394786

[6] Shachar SS Deal AM Weinberg M, . Body composition as a predictor of toxicity in patients receiving anthracycline and taxane-based chemotherapy for early-stage breast cancer. *Clin Cancer Res*. 2017;23(14):3537–43. 28143874 10.1158/1078-0432.CCR-16-2266 PMC5511549

[7] Usiskin I Li F Irwin ML Cartmel B Sanft T. Association between pre-diagnosis BMI, physical activity, pathologic complete response, and chemotherapy completion in women treated with neoadjuvant chemotherapy for breast cancer. *Breast Cancer*. 2019;26(6):719–28. 31119682 10.1007/s12282-019-00974-3

[8] van Waart H Stuiver MM van Harten WH, . Effect of low-intensity physical activity and moderate- to high-intensity physical exercise during adjuvant chemotherapy on physical fitness, fatigue, and chemotherapy completion rates: results of the PACES randomized clinical trial. *J Clin Oncol*. 2015;33(17):1918–27. 25918291 10.1200/JCO.2014.59.1081

[9] An KY Arthuso FZ Kang DW, . Exercise and health-related fitness predictors of chemotherapy completion in breast cancer patients: pooled analysis of two multicenter trials. *Breast Cancer Res Treat*. 2021;188(2):399–407. 33779887 10.1007/s10549-021-06205-8

[10] Velthuis MJ May AM Koppejan-Rensenbrink RA, . Physical Activity During Cancer Treatment (PACT) study: design of a randomised clinical trial. *BMC Cancer*. 2010;10:272. 20534147 10.1186/1471-2407-10-272 PMC2927992

[11] Travier N Velthuis MJ Steins Bisschop CN, . Effects of an 18-week exercise programme started early during breast cancer treatment: a randomised controlled trial. *BMC Med*. 2015;13:121. 26050790 10.1186/s12916-015-0362-z PMC4461906

[12] Witlox L Hiensch AE Velthuis MJ, . Four-year effects of exercise on fatigue and physical activity in patients with cancer. *BMC Med*. 2018;16(1):86. 29879968 10.1186/s12916-018-1075-x PMC5992660

[13] van Waart H Stuiver MM van Harten WH Sonke GS Aaronson NK. Design of the Physical Exercise During Adjuvant Chemotherapy Effectiveness Study (PACES): a randomized controlled trial to evaluate effectiveness and cost-effectiveness of physical exercise in improving physical fitness and reducing fatigue. *BMC Cancer*. 2010;10:673. 21138561 10.1186/1471-2407-10-673 PMC3002358

[14] Longo DL Duffey PL DeVita VT Jr. Wesley MN Hubbard SM Young RC. The calculation of actual or received dose intensity: a comparison of published methods. *J Clin Oncol*. 1991;9(11):2042–51. 1941063 10.1200/JCO.1991.9.11.2042

[15] De Backer IC Schep G Hoogeveen A Vreugdenhil G Kester AD van Breda E. Exercise testing and training in a cancer rehabilitation program: the advantage of the steep ramp test. *Arch Phys Med Rehabil*. 2007;88(5):610–6. 17466730 10.1016/j.apmr.2007.02.013

[16] Bongers BC DE Vries SI Helders PJ Takken T. The steep ramp test in healthy children and adolescents: reliability and validity. *Med Sci Sports Exerc*. 2013;45(2):366–71. 22903141 10.1249/MSS.0b013e31826e32c5

[17] Bongers BC Werkman MS Arets HG Takken T Hulzebos HJ. A possible alternative exercise test for youths with cystic fibrosis: the steep ramp test. *Med Sci Sports Exerc*. 2015;47(3):485–92. 25010405 10.1249/MSS.0000000000000440

[18] Rozenberg R Bussmann JB Lesaffre E Stam HJ Praet SF. A steep ramp test is valid for estimating maximal power and oxygen uptake during a standard ramp test in type 2 diabetes. *Scand J Med Sci Sports*. 2015;25(5):595–602. 25439985 10.1111/sms.12357

[19] Stuiver MM Kampshoff CS Persoon S, . Validation and refinement of prediction models to estimate exercise capacity in cancer survivors using the steep ramp test. *Arch Phys Med Rehabil*. 2017;98(11):2167–73. 28322759 10.1016/j.apmr.2017.02.013

[20] Weemaes ATR Beelen M Bongers BC Weijenberg MP Lenssen AF. Criterion validity and responsiveness of the steep ramp test to evaluate aerobic capacity in survivors of cancer participating in a supervised exercise rehabilitation program. *Arch Phys Med Rehabil*. 2021;102:2150–6. 34023324 10.1016/j.apmr.2021.04.016

[21] Liutkauskiene S Grizas S Jureniene K Suipyte J Statnickaite A Juozaityte E. Retrospective analysis of the impact of anthracycline dose reduction and chemotherapy delays on the outcomes of early breast cancer molecular subtypes. *BMC Cancer*. 2018;18(1):453. 29678165 10.1186/s12885-018-4365-y PMC5910571

[22] Shrier I Platt RW. Reducing bias through directed acyclic graphs. *BMC Med Res Methodol*. 2008;8(1):70. 18973665 10.1186/1471-2288-8-70 PMC2601045

[23] Twisk JWR. *Introduction in Applied Biostatistics*. Amsterdam (the Netherlands): Reed Business Education; 2014.

[24] Campbell KL Winters-Stone KM Wiskemann J, . Exercise guidelines for cancer survivors: consensus statement from International Multidisciplinary Roundtable. *Med Sci Sports Exerc*. 2019;51(11):2375–90. 31626055 10.1249/MSS.0000000000002116 PMC8576825

[25] Cespedes Feliciano EM Chen WY Lee V, . Body composition, adherence to anthracycline and taxane-based chemotherapy, and survival after nonmetastatic breast cancer. *JAMA Oncol*. 2020;6(2):264–70. 31804676 10.1001/jamaoncol.2019.4668 PMC6902178

[26] Courneya KS Segal RJ McKenzie DC, . Effects of exercise during adjuvant chemotherapy on breast cancer outcomes. *Med Sci Sports Exerc*. 2014;46(9):1744–51. 24633595 10.1249/MSS.0000000000000297

[27] Hayes SC Steele ML Spence RR, . Exercise following breast cancer: exploratory survival analyses of two randomised, controlled trials. *Breast Cancer Res Treat*. 2018;167(2):505–14. 29063309 10.1007/s10549-017-4541-9

[28] Kurk S Peeters P Stellato R, . Skeletal muscle mass loss and dose-limiting toxicities in metastatic colorectal cancer patients. *J Cachexia Sarcopenia Muscle*. 2019;10(4):803–13. 31094083 10.1002/jcsm.12436 PMC6711417

[29] Cespedes Feliciano EM Lee VS Prado CM, . Muscle mass at the time of diagnosis of nonmetastatic colon cancer and early discontinuation of chemotherapy, delays, and dose reductions on adjuvant FOLFOX: the C-SCANS study. *Cancer*. 2017;123(24):4868–77. 28881381 10.1002/cncr.30950 PMC5716836

[30] Mijwel S Cardinale DA Norrbom J, . Exercise training during chemotherapy preserves skeletal muscle fiber area, capillarization, and mitochondrial content in patients with breast cancer. *FASEB J*. 2018;32(10):5495–505. 29750574 10.1096/fj.201700968R

[31] Hiensch AE Bolam KA Mijwel S, . Doxorubicin-induced skeletal muscle atrophy: elucidating the underlying molecular pathways. *Acta Physiol (Oxf)*. 2020;229:e13400. 31600860 10.1111/apha.13400 PMC7317437

[32] Mijwel S Bolam KA Gerrevall J Foukakis T Wengstrom Y Rundqvist H. Effects of exercise on chemotherapy completion and hospitalization rates: the OptiTrain breast cancer trial. *Oncologist*. 2020;25(1):23–32. 31391297 10.1634/theoncologist.2019-0262 PMC6964125

[33] Bland KA Zadravec K Landry T Weller S Meyers L Campbell KL. Impact of exercise on chemotherapy completion rate: a systematic review of the evidence and recommendations for future exercise oncology research. *Crit Rev Oncol Hematol*. 2019;136:79–85. 30878132 10.1016/j.critrevonc.2019.02.005

[34] Kirkham AA Gelmon KA Van Patten CL, . Impact of exercise on chemotherapy tolerance and survival in early-stage breast cancer: a nonrandomized controlled trial. *J Natl Compr Canc Netw*. 2020;18(12):1670–7. 33285521 10.6004/jnccn.2020.7603

[35] Gollhofer SM Wiskemann J Schmidt ME, . Factors influencing participation in a randomized controlled resistance exercise intervention study in breast cancer patients during radiotherapy. *BMC Cancer*. 2015;15:186. 25885634 10.1186/s12885-015-1213-1 PMC4466838

