# Supplementary material for: Physical Fitness and Chemotherapy Tolerance in Patients with Early-Stage Breast Cancer
Source: Med Sci Sports Exerc. 2021 Dec 27;54(4):537–42. doi: 10.1249/MSS.0000000000002828 (PMC8920022; doi:10.1249/MSS.0000000000002828)
Supplement: Supplementary file 1 [file msse-54-537-s001.docx]

**Supplemental Digital Content 1: chemotherapy regimens**

| **Abbrevation** | **Agents included** | **Total**  **N= 419** | **%** |
| --- | --- | --- | --- |
| **TAC** | Docetaxel, doxorubin, cyclophoshamide | 139 | 32.9 |
| **FEC or AC** | 5-fluorocacil, epirubicin, cyclophosphamide (FEC), doxorubicin and cyclophosphamide (AC) | 60 | 14.3 |
| **FEC + doce.** | 5-fluorocacil, epirubicin, cyclophosphamide, docetaxel | 98 | 23.3 |
| **AC/EC followed by taxanes (sequential regime)** | Doxorubicin, cyclophosphamide, paclitaxel | 110 | 26.3 |
| **Miscellaneous** | Docetaxel, capecitabine, paclitaxel, epirubicin, cyclophosphamide | 12 | 2.9 |

Description of regimens classified as ‘miscellaneous’:

- Combination therapy docetaxel and capecitabine (n=5)
- Combination therapy of capecitabine and paclitaxel (n=3)
- Monotherapy paclitaxel (n=3)
- Epirubicin, cyclophosphamide and docetaxel, all given sequential (n=1)
